# Berberine inhibits the Warburg effect through TET3/miR-145/HK2 pathways in ovarian cancer cells

**DOI:** 10.7150/jca.48896

**Published:** 2021-01-01

**Authors:** Jie Li, Yuliang Zou, Meili Pei, Yun Zhang, Yu Jiang

**Affiliations:** 1 Department of Pathology, the First Affiliated Hospital of Xi'an Jiaotong University, Xi'an, China.; 2 Department of Gynecology and Obstetrics, the First Affiliated Hospital of Xi'an Jiaotong University, Xi'an, China.

**Keywords:** Berberine, the Warburg effect, methylation, ovarian cancer, miR-145

## Abstract

**Background:** Berberine, as an alkaloid, has a significant antitumor effect, but its mechanism in tumor metabolism, especially the Warburg effect has not been elucidated.

**Objectives:** To study the molecular mechanism of berberine regulating the Warburg effect in ovarian cancer cells.

**Methods:** Treatment by berberine in SKOV3 and 3AO cells or inhibited by miR-145 inhibitor transfection in berberine-treated cells to examine the changes in HK2 expression, glucose consumption and lactate production. The methylation status in the promoter region of pre-miR-145 gene was examined by bisulfite sequencing. Dual-luciferase reporter assay was conducted to verify the direct binding of miR-145 to HK2. Finally, the expression of TET3 in ovarian cancer was investigated by quantitative real-time PCR and immunohistochemistry.

**Results:** We found berberine inhibited the Warburg effect by up-regulating miR-145, miR-145 targeted HK2 directly. Berberine increased the expression of miR-145 by promoting the expression of TET3 and reducing the methylation level of the promoter region of miR-145 precursor gene. We further found that TET3 expression was negatively correlated with clinical stage and pathological grade.

**Conclusions:** Our results revealed berberine increased the TET3-mediated demethylation and promoted the suppression of miR-145 on HK2 to antagonize the Warburg effect of ovarian cancer cells.

## Introduction

The incidence of ovarian cancer, which has the highest mortality rate among gynecological tumors, has little change 1. The data of American surveillance, epidemiology and results (SEER) database shows that the incidence of new cases of ovarian cancer decreased by only 1.9% every year from 2004 to 2013 2. In China, the incidence rate of ovarian cancer has increased by 30% over the past 10 years 3. It is the main direction of ovarian cancer research to study the mechanism of ovarian cancer development, obtain new early diagnosis and treatment targets, and develop targeted drugs.

The Warburg effect, or aerobic glycolysis, is an important energy metabolism characteristic for maintaining the malignant phenotype of tumor cells 4. It is catalyzed by rate limiting enzymes such as hexokinase-2 (HK2), phosphofructokinase-2 (PFK2), pyruvate kinase-2 (PKM2), pyruvate dehydrogenase kinase-1 (PDK1) and lactate dehydrogenase (LDH) 5. The Warburg effect of tumor cells shows the high glycolysis rate of tumor cells 6. It not only provides energy for the growth of tumor cells, but also provides a large number of intermediate products for cell biosynthesis, which provides appropriate energy and nutrition for the rapid proliferation of tumor cells, and helps tumor cells escape from the immune system, increasing their invasive ability 7. Therefore, the key regulatory points of Warburg effect can provide a good direction and strategy for tumor targeted therapy.

microRNA is an important molecule that regulates the physiological and pathological processes, and plays an important role in the process of tumor development 8. microRNAs has been shown to be involved in the Warburg effect of tumors 9. Our previous results indicated that miR-145 expression was reduced in ovarian cancer, and miR-145 could regulate the progression of ovarian cancer by inhibiting the Warburg effect of ovarian cancer cells 10.

Berberine is an alkaloid extracted from coptis, phellodendron and three needles. It has significant antibacterial, antiarrhythmic, antihypertensive and blood lipid regulating effects 11. A large number of studies have shown that berberine has a significant antitumor effect and can participate in the antitumor process of many kinds of cancer, such as liver cancer 12, lung cancer 13, colon cancer 14, esophageal cancer 15, leukemia 16, and so on. Berberine can inhibit tumor progression by inhibiting tumor angiogenesis, promoting apoptosis, blocking invasion and metastasis, reversing drug resistance, etc. 17-20, but its mechanism in tumor metabolism, especially the Warburg effect has not been revealed.

In our study, we found for the first time that berberine can inhibit the Warburg effect of ovarian cancer cells, and berberine increased the expression of miR-145 by promoting the expression of TET3 and reducing the methylation level of the promoter region of miR-145 precursor gene. The regulatory mechanism of berberine/TET3/miR-145/HK2 pathway in the Warburg effect of ovarian cancer cells provides potential therapeutic targets in treatment of ovarian cancer.

## Materials and methods

### Human tissue specimens

Human normal ovarian tissue samples and ovarian carcinomas and were collected from patients at The First Affiliated Hospital of Xi'an Jiaotong University, PR China. This study was approved by the Ethics Committee of The First Affiliated Hospital of Xi'an Jiaotong University, China. The details of tissues are shown in Table 1.

### Cell culture and berberine treatment

The human ovarian cancer cell line SKOV3 was obtained from the Shanghai Cell Bank of Chinese Academy of Sciences (Shanghai, China), 3AO was from the Shandong Academy of Medical Sciences (Jinan, China). Cells were maintained in RPMI 1640 supplemented with 10% newborn bovine serum (GIBCO, Grand Island, 108 NY, USA). The cells were exposure to 40 μM of berberine (for SKOV3) or 80 μM of berberine (for 3AO).

### Quantitative real-time PCR (qRT-PCR)

Methods described in previous studies 10. The primer sequences were showed in Table 2.

### Western blot

The cells were washed with PBS and placed on ice. 100 μL of cell lysate (Roche, Indianapolis, IN, USA) was added into each pore for 10 minutes. The cells were collected by cell scraping and then centrifuged at low temperature (4 °C), 12000 R / min for 20 minutes. After the supernatant was discarded, 5× buffer was added to the EP tube. The protein was denatured at 100 °C for 5 minutes. 12% SDS-PAGE separation gel and 5% upper layer gum were prepared. The total protein of the cells was added to gel electrophoresis. According to the molecular weight, the film was transferred to NC membrane, and 5% of the fresh skim milk was closed, then added with rabbit anti-human TET3 (1:500; Cell Signaling Technology, Danvers, MA, USA), HK2 (1:500; Cell Signaling Technology, Danvers, MA, USA) and mouse anti-human, β-actin (1:1000; Cell Signaling Technology, Danvers, MA, USA). 4 degrees fridge, and the shaking table for the night followed by TBST was used to wash for 5 times, each time for more than 8 minutes, and HRP-conjugated goat anti-rabbit or anti-mouse IgG (1:2000) was added to incubate at room temperature for about 1 h. Similarly, TBST was washed 5 times for ECL chemiluminescence (Pierce, Rockford, IL, USA) by a chemiluminescence imaging system (Bio-Rad, Richmond, CA, USA).

### microRNA mimic or inhibitor transfection

Methods described in previous studies 21.

### siRNA and transient transfection

Human TET3 siRNA was purchased from GenePharma (Shanghai, China). The cells in logarithmic growth stage were inoculated into 6-well plates and transfected when the cells were covered with 40%-50% of the culture plates. The transfection process was carried out according to the instructions of the X-treme GENE siRNA Transfection Reagent (Roche, Indianapolis, IN, USA) following the manufacturer's protocol. The cells were treated 48 hours after transfection, and then the transfection efficiency was verified and the next experiment was carried out.

### CCK8 assay

A single cell suspension containing 10% fetal bovine serum was prepared and inoculated into 96-well plates with 5,000 cells/hole and three multiple holes. After 24, 48, and 72 h of culture, 10 μL CCK8 (7Sea, Shanghai, China) solution was added to each pore, and then incubated for 1.5 h. The absorbance value of each pore (OD 450 nm) was determined using an EnSpire Reader (PerkinElmer, USA), and the growth curve was drawn.

### Measurement of glucose consumption and lactate production

The cells were inoculated into a 6-well culture plate and placed in an incubator for 24 hours at 37 °C and 5% CO2. The glucose concentration and lactate production in the supernatant were measured using a glucose assay kit and a lactate assay colorimetric kit (Nanjing Jiancheng Bioengineering Institute; Nanjing, China) according to the manufacturer's instructions. The results were calculated based on the standard curve and normalized to the cell number.

### Bisulfite sequencing

DNA bisulfite modification and purification were performed using an EZ DNA methylation-Direct kit (ZYMO RESEARCH, CA). The sodium bisulfite-converted DNA was amplified with TaKaRa Taq™Hot Start Version (Takara).

### Luciferase reporter assay

Methods described in previous studies 21.

### Immunohistochemistry

The paraffin embedded tissue sections were dewaxed twice with xylene to be transparent, and then soaked in alcohol for 10 minutes according to the concentration gradient, which were anhydrous alcohol, 95%, 90%, 80%, 70%, 50% alcohol and distilled water. Take out the slices and immerse them in 0.3% methanol-H_2_O_2_ solution, and wash them with PBS 3 times for 3 min at room temperature for 20 min. Immerse the slide in the antigen repair buffer (citric acid) and boil it under high pressure in a pressure cooker for 10 minutes, then let the water temperature cool naturally. PBS was washed 3 times, 3 minutes each time. Goat serum was used for 20 minutes. Draw a circle around the tissue with a crayon, dilute an antibody according to the proportion and drop it into the circle, slice it into a wet box, and incubate it overnight at 4 °C. The slices were washed by PBS for 5 min × 3 times. The HRP-labeled secondary antibody (MaxVision HRP-Polymer anti-Mouse/Rabbit IHC Kit, Maixin Biotech Corp, Fuzhou, China) was diluted in proportion and dripped onto the tissue, incubated at room temperature for 30 min, washed with PBS for 5 min × 3 times. Streptavidin peroxidase was added and incubated at room temperature for 30 min, washed with PBS for 5 min × 3 times. DAB solution was dripped, and the color development was controlled under microscope. PBS was washed for 5 min × 3 times. Hematoxylin was dripped into the slices and then rinsed for 10 s. The slices were soaked in alcohol (50%, 70%, 80%, 90%, 95%, 100%) and then immersed in xylene until transparent. The transparent tissue sections were dripped with neutral gum and sealed with cover glass. Digital images were acquired on an Olympus BH-2 microscope (Olympus, Tokyo, Japan) installed with a DeltaPix Camera and software (DeltaPix, Maalov, Denmark). For statistical analysis, the intensity of staining was obtained by 2 pathologists. Intensity was semiquantitatively scored as weak (1 point), moderate (2 points), or strong (3 points). For an individual case, the immunohistochemical composite score was calculated based on the extent multiplied by the intensity score.

### Statistical analysis

Data were analyzed using SPSS 22.0 software (Chicago, IL). Differences were considered significant at *p* < 0.05 (*) or highly significant at *p* < 0.001 (**).

## Results

### Berberine inhibited the Warburg effect by up-regulating miR-145 in ovarian cancer cells

**Figure 1:**
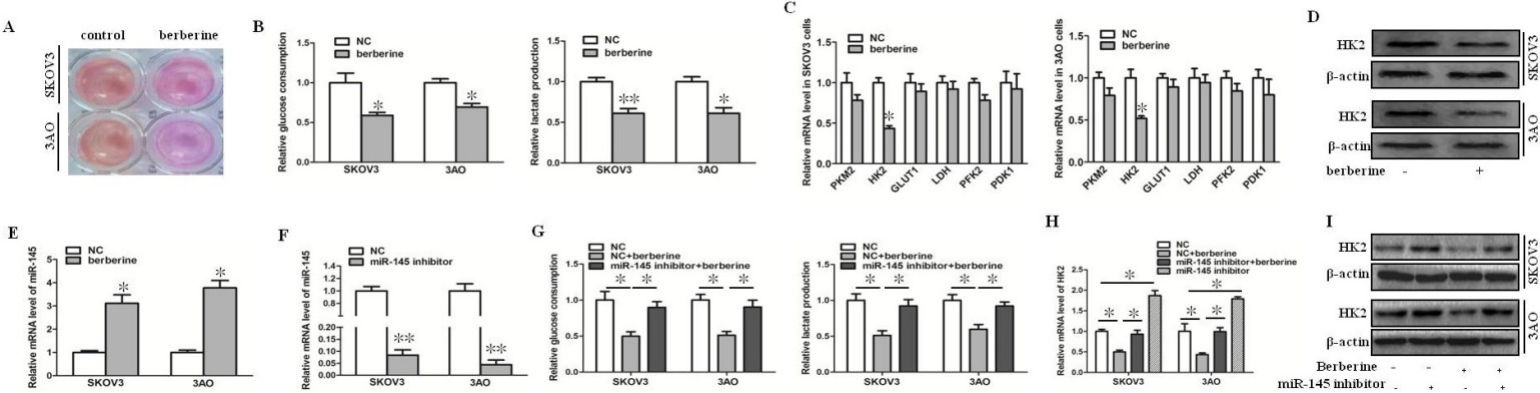
** Berberine inhibited the Warburg effect by up-regulating miR-145 in ovarian cancer cells.** (**A**) After treatment of berberine, acidification of the culture medium was evaluated by visually inspecting the color of the medium. (**B**) Effects of berberine on glucose uptake and lactate production in SKOV3 and 3AO cells. (**C**) Detection of the effect of berberine on the mRNA expression of glycolysis related enzymes by qRT-PCR. (**D**) Westernblot results showed that berberine could inhibit the protein level of HK2. (**E**) Berberine promoted the expression of miR-145 in ovarian cancer cells. (**F**) The expression of miR-145 after transfection miR-145 inhibitor. (**G**) Knockdown of miR-145 reversed the inhibitory effect of berberine on glucose consumption and lactate production in SKOV3 and 3AO cells. (**H**) qRT-PCR results showed downexpression of miR-145 reversed berberine's inhibition of HK2 expression. (**I**) Western blot results showed down-expression of miR-145 reversed berberine's inhibition of HK2 expression. PKM2, pyruvate kinase M2; HK2, Hexokinase2; GLUT1, glucose transporter 1; LDH, lactate dehydrogenase; PFK2, phosphofructokinase 2; PDK1, pyruvate dehydrogenase kinase. *P < 0.05, **P < 0.01, t test.

We found that after treatment of SKOV3 and 3AO cells with berberine, the color of the culture medium was redder than that of the control group (Fig. 1A), indicating that after treatment with berberine, the alkalinity of the culture medium was higher than that of the control group, suggesting that the amount of lactate production was reduced, so we speculated that berberine could inhibit the Warburg effect of ovarian cancer cells. Then, we detected glucose consumption and lactate production after treatment of SKOV3 and 3AO cells with berberine for 24 hours. The results showed that both glucose consumption and lactate production decreased after berberine treatment (Fig. 1B). Furthermore, the effect of berberine on glycolysis related enzymes was detected, the results of qRT-PCR and westernblot suggested that berberine could significantly down regulate the expression of HK2 (Fig. 1C,D).

Our previous results confirmed that miR-145 could target HK2 to inhibit the Warburg effect of ovarian cancer cells 10. In order to explore whether miR-145 participates in Warburg effect of ovarian cancer cells regulated by berberine, we first detected the change of miR-145 level after treatment of berberine. The results showed that berberine promoted the expression of miR-145 (Fig. 1E). Knockdown of miR-145 (Fig. 1F) reversed the inhibition of berberine on glucose consumption and lactate production (Fig. 1G). The results of qRT-PCR and westernblot showed that berberine inhibited the expression of HK2, and the inhibition was blocked by knocking down of miR-145 (Fig. 1H, I). In conclusion, berberine could inhibit the expression of HK2 through miR-145, thus inhibiting the Warburg effect of ovarian cancer cells.

### Berberine increased the expression of miR-145 by promoting the expression of TET3 and reducing the methylation level of the promoter region of miR-145 precursor gene

**Figure 2:**
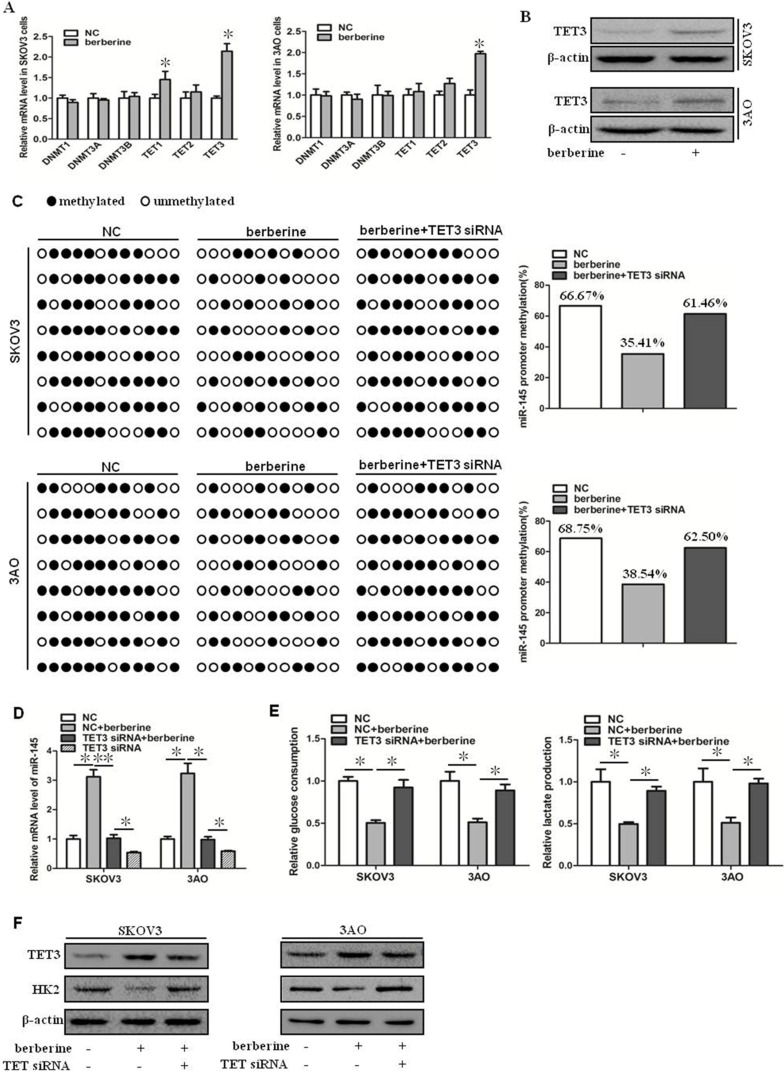
** Berberine increased the expression of miR-145 by promoting the expression of TET3 and reducing the methylation level of the promoter region of miR-145 precursor gene.** (**A**) qRT-PCR showed that TET3 was significantly increased at mRNA level in cells treated by berberine in SKOV3 and 3AO cells. (**B**) Western blot showed that TET3 was significantly increased at protein level in cells treated by berberine. (**C**) BSP results showed berberine decreased the methylated proportion in the promoter region of the miR-145, TET3 downxpression reversed the methylation effect of berberine. (**D**) Knockdown of TET3 weakened the promotion of berberine on the expression of miR-145. (**E**) Knockdown of TET3 blocked the inhibition of berberine on glucose consumption and lactate production. (**F**) Western blot results showed berberine inhibited the expression of HK2, which was reversed when TET3 was knocked down. *P < 0.05, **P < 0.01, *t* test.

Berberine can upregulate the expression level of miR-145, but the specific mechanism is not clear. It has been reported that the expression of miR-145 is regulated by methylation 10. We speculated that berberine might increase the expression of miR-145 by reducing the methylation level of the promoter region of miR-145 precursor gene. We first examined the expression of DNMT and TET family members in berberine-treated ovarian cancer cells. As shown by qRT-PCR results (Fig. 2A). TET3 was significantly upregulated, while other expression levels were not significantly changed. TET3 protein level was then detected, and consisted with mRNA reduction, TET3 protein level was increased in berberine-treated ovarian cancer cells (Fig. 2B). Hence, the effect of TET3 on the level of miR-145 was further examined. Down-expression of TET3 reversed the berberine-restrained methylation in the promoter region of miR-145 in both SKOV3 and 3AO cells (Fig. 2C), resulting in the downregulation of miR-145 by TET3 downexpression in berberine-treated cells (Fig. 2D). Consequently, downexpression of TET3 antagonized the decrease in glucose consumption and lactate production (Fig. 2E) and HK2 expression (Fig. 2F). These results indicated that miR-145 expression was positively influenced by TET3-mediated DNA demethylation, which was the key mechanism manipulated by berberine to promote miR-145-antagonized the Warburg effect.

### miR-145 inhibited downexpression of TET3-promoted the Warburg effect

**Figure 3:**
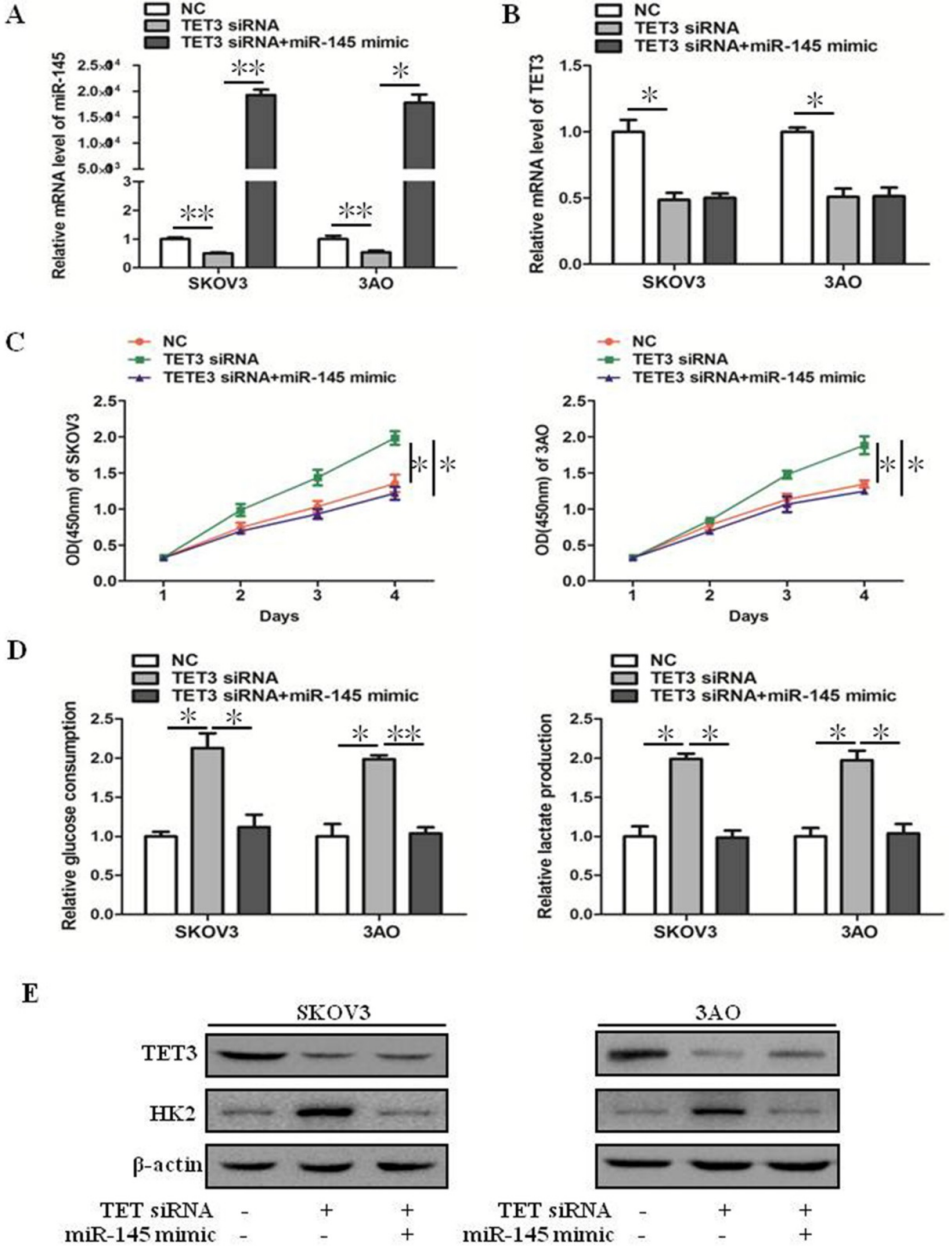
** miR-145 inhibited downexpression of TET3-promoted the Warburg effect.** (**A**) qRT-PCR showed that TET3 downexpression downregulated miR-145 expression which was recovered by miR-145 mimic transfection. (**B**) qRT-PCR showed that ectopic expression of miR-145 had negligible effect on TET3 expression in TET3-knockdown cells. (**C**) Promotion of cell growth caused by TET3 siRNA was alleviated by miR-145 mimic, especially after 3 days of transfection of miR-145 mimic. (**D**) Knockdown of TET3 promoted glucose consumption and lactate production in SKOV3 and 3AO cells, which was blocked by overexpression of miR-145. (**E**) Western blot results showed overexpression of miR-145 counteracted the promotion of HK2 expression by knockdown of TET3. *P < 0.05, **P < 0.01, *t* test.

Downexpression of TET3 inhibited the mRNA level of miR-145 (Fig. 3A), but ectopic expression of miR-145 in TET3-downxpressed cells did not affect TET3 expression (Fig. 3B), these results indicated that TET3 was the upstream regulator of miR-145. Down-expression of TET3 promoted cell growth, glucose consumption and lactate production in ovarian cancer cells, which was reversed by overexpression of miR-145 (Fig. 3C, D). Accordingly, the increase of HK2 expression induced by TET3 knockdown was reversed by overexpression of miR-145 (Fig. 3E). In general, TET3 inhibited theWarburg effect through regulating miR-145.

### miR-145 targeted HK2 directly

**Figure 4:**
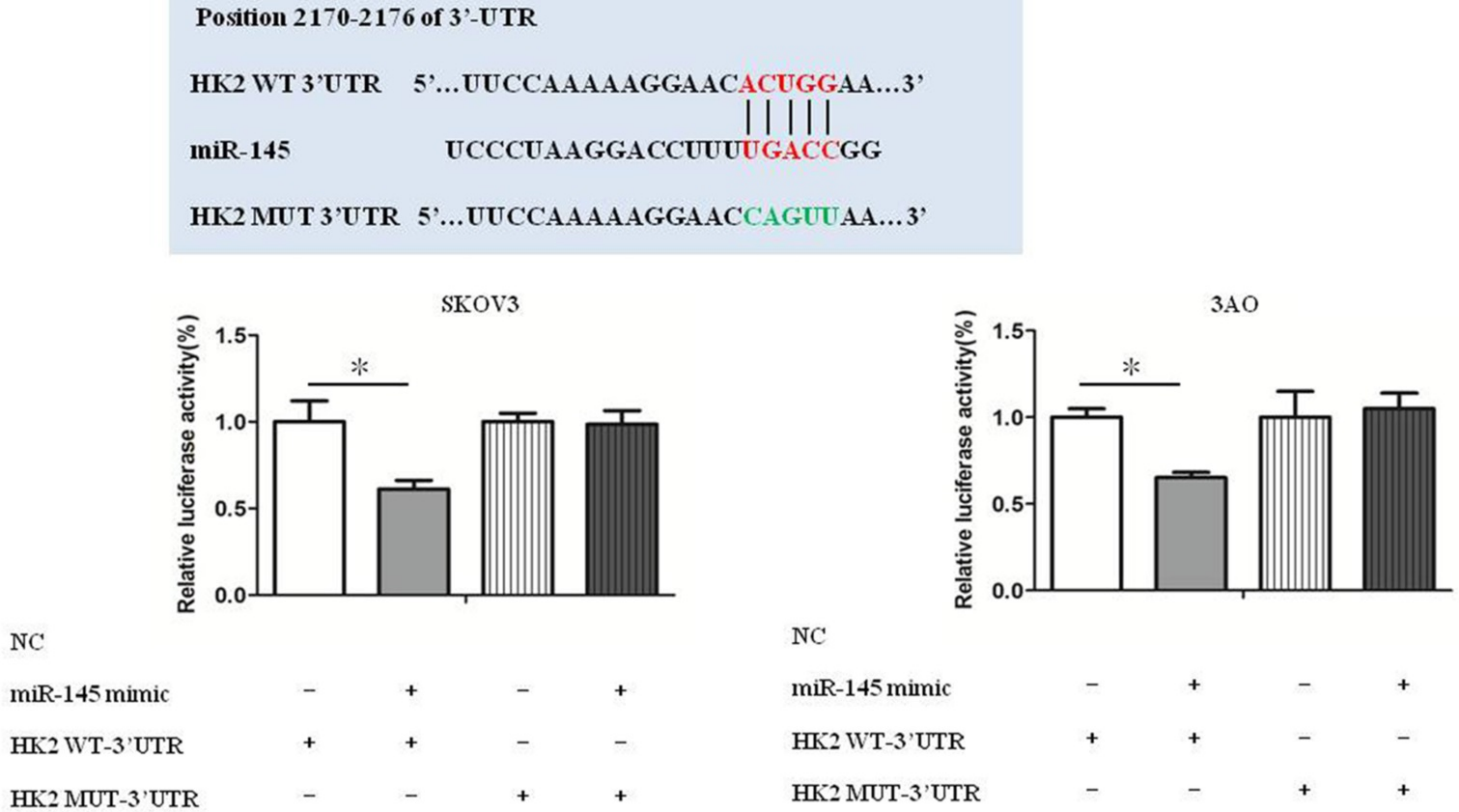
** miR-145 targeted HK2 directly.** Luciferase reporter assays show that HK2 was a target gene of miR-145. *P < 0.05, **P < 0.01, *t* test.

Luciferase reporter assays show that HK2 was a target gene of miR-145 (Fig. 4). Our previous studies have confirmed that miR-145 inhibits Warburg effect by targeting HK2 in ovarian cancer cells 10.

### The expression of TET3 was decreased in ovarian cancer tissues

**Figure 5:**
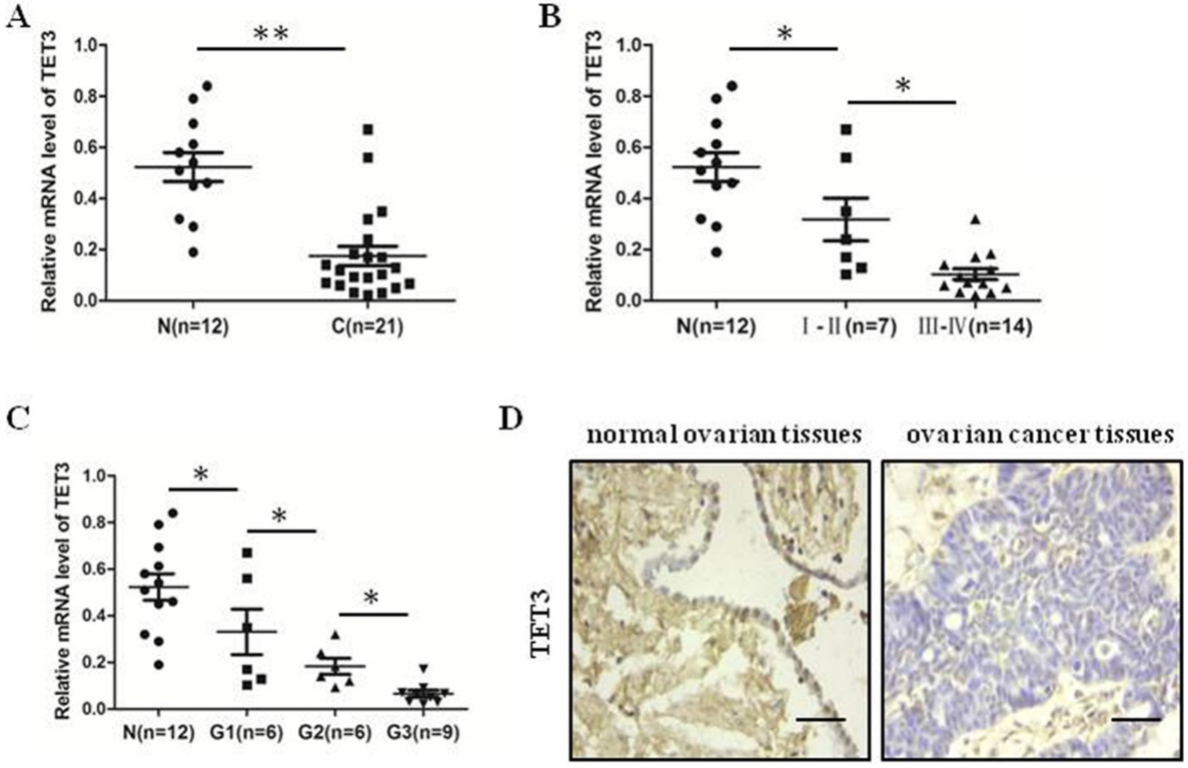
** The expression of TET3 was decreased in ovarian cancer tissues.** (**A**) The expression level of TET3 in ovarian cancer tissues was lower than that in normal ovarian tissues. (**B**) The expression level of TET3 was negatively correlated with the clinical stage of ovarian cancers. (**C**) The expression level of TET3 was negatively correlated with the pathological grade of ovarian cancers. (**D**) Immunohistochemical results showed the expression level of TET3 in ovarian cancer tissues and normal ovarian tissues. n, normal ovarian tissues, c, ovarian cancer tissues, Scale bar, 100 µm, *P < 0.05, **P < 0.01, *t* test.

We detected mRNA level of TET3 in 21 ovarian cancer tissues and 11 normal ovarian tissues by quantitative real-time PCR analysis. We identified that TET3 level in ovarian cancer tissues were lower than in normal ovarian tissues (Fig. 5A). Moreover, the results showed the more late the clinical period, the lower the expression of TET3 (Fig. 5B), and the results demonstrated that the expression of TET3 was inversely associated with the grade of differentiation of malignant cells (Fig. 5C). Typical immunohistochemistry (IHC) photographs from both ovarian cancer and normal ovarian groups are shown in Fig. 5D. Clinicopathological correlation analysis of TET3 level to ovarian cancer showed that the immunohistochemical composite score of TET3 was negatively associated with grade of differentiation of malignant cells (Table 3).

## Discussion

**Figure 6:**
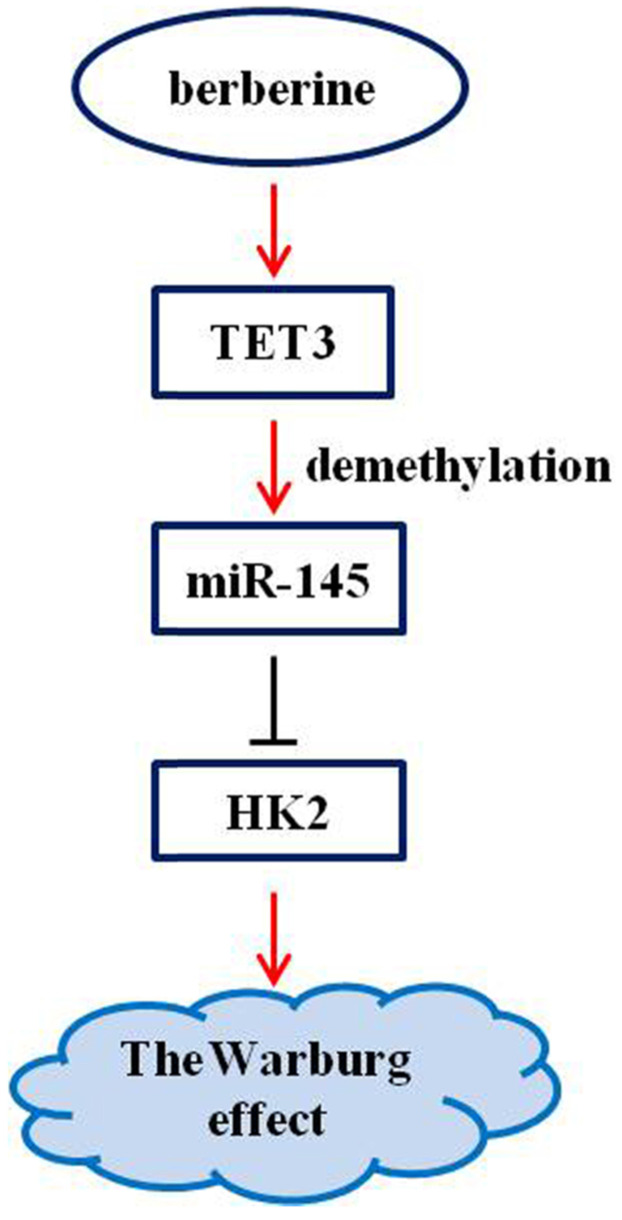
Schematic representation of the anti-Warburg effect mechanism of berberine.

Berberine has a clear antitumor activity 17-20. It can participate in the antitumor process by regulating autophagy, inhibiting proliferation and promoting apoptosis, reversing drug resistance, inhibiting tumor cell invasion and metastasis, anti-angiogenesis and other mechanisms 17-20. Here, we first reported that the pathway composed of TET3, miR-145, and HK2 was implicated in the mechanism of berberine to inhibit the Warburg effect (Figure 6), and that the demethylation of miR-145 by TET3 played an important role in repression of the Warburg effect.

Berberine is an alkaloid extracted from coptis, phellodendron and three needles. It has significant antibacterial, antiarrhythmic, antihypertensive and blood lipid regulating effects 11. The role of berberine in ovarian cancer has been gradually discovered. Liu et al. 22 found berberine in combination with cisplatin induced necroptosis and apoptosis in ovarian cancer cells. Hou et al. 23 demonstrated berberine induced oxidative DNA damage and impaired homologous recombination repair in ovarian cancer cells. In addition, studies have shown that berberine sensitized ovarian cancer cells to cisplatin through miR-21/PDCD4 axis 24. Collectively, these findings, together with our data, improved the mechanism of berberine against ovarian cancer. Nevertheless, the pathway relative to the entrance of the berberine into cells and the direct targets of the berbreine merits further study.

The Warburg effect can not only provide the precursor of macromolecule synthesis and metabolism for the rapid growth of tumor cells, but also create a suitable microenvironment to provide growth advantages for tumor cells 25, 26. Our study found that berberine could inhibit the Warburg effect of ovarian cancer cells, and the inhibition effect was achieved by promoting the expression of miR-145. Combined with our previous studies, we clarified that miR-145 targeted HK2 to inhibit the Warburg effect of ovarian cancer cells, and revealed that berberine/miR-145/HK2 plays an important role in the Warburg effect of ovarian cancer cells.

DNA methylation is closely related to the occurrence and development of tumor. It has been admitted that abnormal DNA methylation/demethylation is a hallmark of cancer 27, 28. In addition to DNMTs, TETs are novel regulators of DNA methylation/demethylation status. Growing evidences suggests that TET-mediated DNA demethylation takes part in tumor development and progression 29, 30. In our study, we found that TET3 was under-expressed in ovarian cancer and negatively correlated with clinical stage and pathological grade. Berberine increased the expression of miR-145 by promoting the expression of TET3 and reducing the methylation level of the promoter region of miR-145 precursor gene. Recent studies showed that TETs were direct targets of multiple microRNAs 31-34; however, there are few studies on the regulation of microRNAs by TETS. In the present study, we found that TET3 could not only promote the expression of miR-145, but also inhibit the Warburg effect of ovarian cancer cells through miR-145. Studies on the effects of TET3 and the Warburg effect have never been reported before.

## Conclusions

In conclusion, we found that berberine inhibited the Warburg effect via promoting TET3-mediated demethylation of pre-miR-145 to promote inhibition of miR-145 on HK2. The study provided the new mechanistic evidence about TET3/miR-145/HK2 pathway in the anti-Warburg effect of berberine, providing evidence for berberine to become a candidate drug for clinical treatment of ovarian cancer.

## Figures and Tables

**Table 1:** Summary of clinical characteristics of patients in the present study

Characteristics	Cases
**Normal ovarian tissues**	
Total number	12
Median age years (range)	52 (46-69)
**Ovarian cancer tissues**	
Total number	21
Median age years (range)	61 (39-72)
**FIGO stage**	
I/II	7
III/IV	14
**Subtypes of ovarian cancer**	
Serous cancer	15
Mucinous cancer	4
Endometrioid cancer	1
Clear cell cancer	1

FIGO, International Federation of Gynecology and Obstetrics.

**Table 2:** Primer sequences for real-time PCR

Genes	Primer sequences(5'-3')
DNMT1	F: CCTGAGGCCTTCACGTTCAA
	R: ACTTGTGGGTGTTCTCAGGA
DNMT3A	F: TATTGATGAGCGCACAAGAGAGC
	R: GGGTGTTCCAGGGTAACATTGAG
DNMT3B	F: GGCAAGTTCTCCGAGGTCTCTG
	R: TGGTACATGGCTTTTCGATAGGA
HK2	F: AAGGCTTCAAGGCATCTG
	R: CCACAGGTCATCATAGTTCC
LDH	F: GGCCTGTGCCATCAGTATCT
	R: GGAGATCCATCATCTCTCCC
PKM2	F: TCCGGATCTCTTCGTCTTTG
	R: GTCTGAATGAAGGCAGTCCC
PFK2	F: GCTATGAAACCAAAACCCCA
	R: TAACGATCAGAGTCGGGGAG
PDK1	F: CAACAGAGGTGTTTACCCCC
	R: ATTTTCCTCAAAGGAACGCC
TET1	F: CCCGAATCAAGCGGAAGAATA
	R: TACTTCAGGTTGCACGGT
TET2	F: CTTTCCTCCCTGGAGAACAGCTC
	R: TGCTGGGACTGCTGCATGACT
TET3	F: GTTCCTGGAGCATGTACTTC
	R: CTTCCTCTTTGGGATTGTCC
β-actin	F: TCCCTGGAGAAGAGCTACGA
	R: AGCACTGTGTTGGCGTACAG

**Table 3:** Clinicopathological correlation of TET3 to ovarian cancer

Clinicopathological parameters of ovarian cancer	Immunohistochemical composite scores		*P* value^a^
	Mean	SD	
**Stage**			0.036*
I/II (n=7)	0.76	0.27	
III/IV (n=14)	0.57	0.15	
**Grade**			0.027*
G1 (n=6)	0.81	0.66	
G2 (n=6)	0.59	0.23	
G3 (n=9)	0.41	0.39	

*P <0.05; ^a^*t* test or one-way ANOVA (two-tailed).
